# Efficient Hydrogen Generation and Total Nitrogen Removal for Urine Treatment in a Neutral Solution Based on a Self-Driving Nano Photoelectrocatalytic System

**DOI:** 10.3390/nano11112777

**Published:** 2021-10-20

**Authors:** Pengbo Wang, Jinhua Li, Yang Xu, Changhui Zhou, Yan Zhang, Lina Zha, Bo Zhang, Jing Bai, Baoxue Zhou

**Affiliations:** 1 Key Laboratory of Thin Film and Microfabrication Technology (Ministry of Education), School of Environmental Science and Engineering, Shanghai Jiao Tong University, Shanghai 200240, China; tianliangjue@sjtu.edu.cn (P.W.); xy0122@sjtu.edu.cn (Y.X.); zhouchanghui-sjtu@sjtu.edu.cn (C.Z.); yan-zhang@sjtu.edu.cn (Y.Z.); zhalina_V5@sjtu.edu.cn (L.Z.); zhangb229@sjtu.edu.cn (B.Z.); bai_jing@sjtu.edu.cn (J.B.); 2 Shanghai Institute of Pollution Control and Ecological Security, Shanghai 200092, China

**Keywords:** nano photoelectrocatalysis, urine, hydrogen generation, self-driving, cyclic catalysis of Cl^−^/Cl•, total nitrogen removal

## Abstract

Urine is the main source of nitrogen pollution, while urea is a hydrogen-enriched carrier that has been ignored. Decomposition of urea to H_2_ and N_2_ is of great significance. Unfortunately, direct urea oxidation suffers from sluggish kinetics, and needs strong alkaline condition. Herein, we developed a self-driving nano photoelectrocatalytic (PEC) system to efficiently produce hydrogen and remove total nitrogen (TN) for urine treatment under neutral pH conditions. TiO_2_/WO_3_ nanosheets were used as photoanode to generate chlorine radicals (Cl•) to convert urea-nitrogen to N_2_, which can promote hydrogen generation, due to the kinetic advantage of Cl^−^/Cl• cyclic catalysis. Copper nanowire electrodes (Cu NWs/CF) were employed as the cathode to produce hydrogen and simultaneously eliminate the over-oxidized nitrate-nitrogen. The self-driving was achieved based on a self-bias photoanode, consisting of confronted TiO_2_/WO_3_ nanosheets and a rear Si photovoltaic cell (Si PVC). The experiment results showed that hydrogen generation with Cl• is 2.03 times higher than in urine treatment without Cl•, generating hydrogen at 66.71 μmol h^−1^. At the same time, this system achieved a decomposition rate of 98.33% for urea in 2 h, with a reaction rate constant of 0.0359 min^−1^. The removal rate of total nitrogen and total organic carbon (TOC) reached 75.3% and 48.4% in 2 h, respectively. This study proposes an efficient and potential urine treatment and energy recovery method in neutral solution.

## 1. Introduction

Nitrogen pollution is the main cause of water eutrophication [1]. As high-concentration nitrogen-containing wastewater, billions of tons of urine are discarded every year, endangering human health [2,3]. Currently, urine contributes to 80% of the nitrogen and 10% of the COD load in municipal sewage [4,5]. Severe nitrogen overload has become the biggest challenge for existing wastewater treatment plants [6]. In order to meet the discharge standard of nitrogen and COD, a series of techniques are adopted including A^2^O, chemical treatment and expanded hydraulic stay time [7], which greatly rise treatment time, floor area, and energy consumption. On the other hand, urea is an abandoned hydrogen source that has been ignored [8,9,10,11]. The thermodynamic decomposition of urea is only 39.95 kJ mol^−1^, which is 1/12 of water splitting 474.38 kJ mol^−1^ (Equations (1) and (2)), indicating that urea is a suitable substitute for water splitting to produce H_2_ [12,13,14]. Therefore, decomposition of urea to H_2_ and simultaneous total nitrogen (TN) removal is an appealing solution for urine treatment [15].

2H_2_O → 2H_2_ + O_2_            ΔG^0^_1_ = +474.38 kJ mol^−1^ (1)

CO(NH_2_)_2_ + H_2_O → CO_2_ + N_2_ + 3H_2_   ΔG^0^_2_ = +39.95 kJ mol^−1^ (2)

To solve the above problem, photoelectrocatalytic technology is a suitable method for processing urine [2]. However, the conversion of urea to N_2_ is a kinetic inert step with a high overpotential. Urea is more easily over-oxidized to nitrate, making it difficult to remove total nitrogen (TN), and available treatment technologies of nitrate are inefficient and expensive [16,17]. Moreover, urea oxidation reactions require strong alkaline conditions in most current research, which hinders the practical application of urine electrochemical treatment, due to faintly acidic actual urine, and alkali cost. Thus, transforming urea into N_2_ while simultaneously producing H_2_ in neutral solution is a challenging issue. Recently, chlorine radicals (Cl•) have been considered as effective oxidant for the treatment of NH_4_^+^ and amine wastewater, since it preferably reacts with electron-rich moieties through one-electron oxidation [18,19,20,21,22]. It has been confirmed that Cl• can selectively react with ammonium or amino to form the main product N_2_, reducing the accumulation of nitrate and nitrite [23,24,25]. Chloride ions (Cl^−^), as a common ion in urine, can be oxidized to Cl• through strongly oxidative photogenerated holes under illumination [26]. Cl• converts urea to N_2_, with the formation of Cl^−^, which is oxidized into Cl• cyclically. Hydrogen generation is promoted since Cl^−^/Cl• cyclic catalysis has a kinetic advantage with a single-electron pathway, compared to direct urea oxidation. More importantly, there is no need to add additional alkali. Thus, the Cl• generation reaction was introduced into this paper to remove TN for the urine treatments, and to generate H_2_ simultaneously.

Copper foam (CF) is considered to be a suitable cathode substrate aiming to produce H_2_, due to its large specific surface area, low lost, excellent conductivity, and stability [27,28,29,30]. CF can be improved to obtain a copper nanowire electrode (Cu NWs/CF) [31], which has a larger specific surface area, and an excellent rapid removal effect on nitrate and nitrite in a solution as a copper-based catalyst [32]. Subsequently, an improved Cu NWs/CF with more abundant active catalytic sites was used as cathode in this paper for H_2_ generation, and nitrate-nitrogen (NO_3_^−^-N) removal.

Inspired by these points, a self-driving nano photoelectrocatalytic (PEC) system was developed to efficiently produce H_2_ and remove TN for urine treatment under neutral pH conditions. TiO_2_/WO_3_ nanosheets, with high catalytic activity, stabilization, and visible-light response due to the lattice matching and energy band alignment between WO_3_ and TiO_2_ [33], was used as photoanode to generate Cl• and HO• [34] for the conversion of urea-nitrogen (Urea-N) to N_2_, and the enhancement of hydrogen generation [35], respectively. Cu NWs/CF were employed as the cathode to produce H_2_ and simultaneously eliminate the over-oxidized nitrate-nitrogen. The impressive results in H_2_ generation and TN removal also benefitted from the design of a self-bias photoanode, which consisted of confronted TiO_2_/WO_3_ nanosheets and a rear Si photovoltaic cell (Si PVC), in which the TiO_2_/WO_3_ mainly absorbed short-wavelength light and generated electron/hole pairs, and the Si PVC captured the filtered long-wavelength light and caused photovoltage, to highly promote the separation of photogenerated charges of the PEC system without the use of external voltage [36]. The results proved the synergistic effect of urea oxidation and H_2_ generation. Additionally, this PEC system could achieve rapid TN removal from urine, indicating that the system could realize urine rapid purification and energy recovery. Hence, this work proposes an efficient and promising method for urine H_2_ generation and TN removal.

## 2. Materials and Methods

### 2.1. Material and Chemicals

The reagents were purchased from China Sinopharm Chemical Reagent Co., Ltd. (Shanghai, China), and all chemicals were analytical grade. Milli-Q ultrapure water system was used to prepare deionized water. Si PVC was obtained from Suzhou XuNing Co., Ltd. (Suzhou, China). Fluorine-doped tin oxide (FTO) glass (13 Ω cm^−1^) was ordered from Nippon Sheet Glass Co., Ltd. (Tokyo, Japan). The CF (3 mm thickness) used as the substrate was purchased from Suzhou Taili Metal Foam Co., Ltd. (Suzhou, China).

### 2.2. Preparation of the Electrodes

Preparation of the WO_3_ nanosheets photoanode referred to the method found in previous research [37,38,39]. The TiO_2_/WO_3_ nanosheets photoanode was prepared through a hydrothermal method, on the basis of the WO_3_ photoanode [33]. During the process, the pre-prepared WO_3_ electrode was first placed aslant in an aqueous solution containing 10 mmol L^−^^1^ ammonium fluorotitanate and 75 mmol L^−^^1^ boric acid, with a constant temperature of 40 °C for 8 h, and the TiO_2_/WO_3_ photoanode was subsequently prepared after a rinsing and drying process. A Si PVC rear photoanode was connected in series with the front TiO_2_/WO_3_ photoanode, and sealed by epoxy resin.

The Cu NWs/CF was synthesized in the following successive sequence: alkaline oxidation [40], high temperature dehydration, and electroreduction methods [31]. In detail, a piece of CF (2 cm × 5 cm) was first placed in an aqueous solution containing 2.5 mol L^−^^1^ sodium hydroxide and 0.125 mol L^−^^1^ sodium persulfate for 4 min to obtain Cu(OH)_2_ nanowire electrodes. Secondly, a pre-prepared Cu(OH)_2_ nanowire electrode was annealed at 180 °C for 180 min to obtain CuO nanowire electrodes. Thirdly, the CuO nanowire electrode was reduced in a typical three-electrode system for 30 min at −1 V (vs. Ag/AgCl) with 1 mol L^−^^1^ Na_2_SO_4_. After washing and drying, a Cu NWs/CF electrode was finally prepared.

### 2.3. Experimental Setup

The reactor of the PEC system in this study was a single chamber reactor under a simulated solar light (100 mW cm^−2^), using a 350 W Xe lamp (Perfect, Shanghai, China). The electrolyte contained 50 mmol L^−^^1^ Na_2_SO_4_, a certain concentration of urea, and a certain concentration of Cl^−^, with a volume of 25 mL. The immersion area of the TiO_2_/WO_3_ photoanode and the copper nanowire counter electrode was 4 cm^2^ (2 cm × 2 cm), and the distance between the electrodes was 1.5 cm. The composition of synthetic urine is described in Table S1. Actual urine was obtained from 5 healthy volunteers. An electrochemical workstation was connected between the photoanode and the counter electrode to monitor the current, and to regulate voltage. Samples were taken out every 30 min for component analysis during the two-hour reaction process. The TN removal rate was calculated by dividing the final TN concentration by the initial TN concentration. All experiments were repeated at least three times to ensure the credibility of the data and results.

### 2.4. Analytical Methods

A scanning electron microscope (SEM) (SUPRA55-VP, Zeiss, Oberkochen, Germany) equipped with an X-ray energy dispersive spectrometer was used to scan the surface of electrodes. Phase composition of electrodes were investigated through X-ray diffraction (XRD, D-Max B, Rigaku, Tokyo, Japan) and X-ray photoelectron spectroscopy (XPS, EA125, Omicron, Vienna, Austria), using XPSPEAK to fit XPS data. The absorption spectra were measured using a UV-visible spectrophotometer (TU-1901, Pgeneral, Beijing, China). A UV-visible spectrophotometer (TU-1810, Persee, Beijing, China) was used to monitor the concentration of ammonia-nitrogen in the solution at 420 nm, colored using potassium sodium tartrate and Nessler reagent after diluting 10 times. Ion chromatography (ICS-1000, Dionex, San Francisco, CA, USA) was used to determine nitrate-nitrogen and nitrite-nitrogen (NO_2_^−^-N) content. The conditions of ion chromatography were as follows: the sample solution was purified by an IC Na column, and the chromatographic column was an IonPac AS23 4 mm × 250 mm ion exchange column with 5.0 mmol/L Na_2_CO_3_ and 0.2 mmol/L NaHCO_3_ eluent, at a flow rate of 1.0 mL/min. The concentration of urea was detected through the high-performance liquid chromatography method (HPLC-2010Plus, Shimadzu, Osaka, Japan). The conditions of HPLC were as follows: chromatographic column: Kromasil 100-NH_2_ column (250 mm × 4.6 mm, 5 μm); eluent: acetonitrile-water (90:10); flow rate: 1.0 mL/min; column temperature: 30°. The electrochemical workstation was used to perform linear sweep voltammetry (LSV) (sweep speed 0.02 V/s), electrochemical impedance spectroscopy, and test the photoelectric performance of the prepared photoanode and monitor the system current. The concentration of total organic carbon (TOC) and TN were monitored by a TOC/TN analyzer (Multi-3100, Analytikjena, Jena, Germany). The type of free radicals was tested using electron spin resonance spectrometer (EPR, Bruker, Billerica, MA, USA) [41]. H_2_ generation was determined using PerfectLight Labsolar 6A (PerfectLight, Beijing, China) and Fuli GC9790 Plus (Fuli, Taizhou, China).

## 3. Results and Discussion

### 3.1. Characterization of the Electrodes

**Figure 1:**
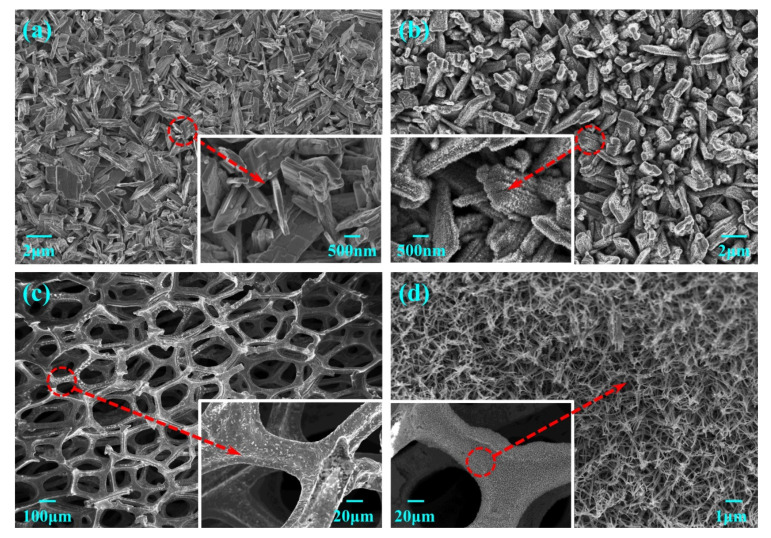
SEM images of the (**a**) WO_3_; (**b**) TiO_2_/WO_3_; (**c**) CF; and (**d**) Cu NWs/CF electrodes.

Figure 1a,b shows the SEM images of the WO_3_ nanosheets and the TiO_2_/WO_3_ nanosheets electrode, respectively. The WO_3_ nanosheets were vertically and crosswise grown on the entire FTO substrate. The size of each WO_3_ nanosheet was approximately 2 μm × 1.5 μm × 0.4 μm. Similar frameworks were observed in WO_3_ and TiO_2_/WO_3_. There appears no significant morphological change to the nanosheet after modification with TiO_2_, but small thorns can be observed, suggesting the successful growth of TiO_2_. TiO_2_ thorns were densely and evenly grown on the entire surface of the WO_3_ nanosheets, which protected and passivated WO_3_. Accordingly, the modified TiO_2_ layer not only increased the photo-response current but also enhanced the stability of WO_3_, due to surface passivation (Figure S1). In addition, the light absorption spectrum of TiO_2_/WO_3_ slightly expanded from 407 nm to 417 nm, compared to the bare WO_3_ (Figure S2), indicating a better absorption of visible light. The phase composition of the samples was investigated using XRD method, which verified the formation of WO_3_ on FTO, whereas no typical peaks of TiO_2_ were detected, due to the thin TiO_2_ layer (Figure S3a). The characteristic peaks of WO_3_ corresponded with a monoclinic crystalline phase (JCPDS no. 43-1035), which was verified to have an excellent photoelectric performance in comparison to other crystalline phases, due to high activity and stability [42]. XPS analysis further demonstrated the existence of TiO_2_ according to the typical peaks at 459.27 eV and 464.96 eV, which are assigned to Ti2p_3/2_ and Ti2p_1/2_, respectively (Figure S3b) [43].

Figure 1c,d shows the SEM images of the CF and the Cu NWs/CF electrodes, respectively. The CF has a frame structure of 400 μm micro-pores in diameter. A smooth surface was seen on the bare CF, which limited the specific surface area; in contrast, the surface became rough and porous after surface treatment (Figure 1d). A large number of nanowire structures were grown resembling a bird’s nest throughout the whole surface of the CF, showing a larger specific surface area, and the diameter of the nanowires was approximately 150 nm, according to the TEM image of Figure S4. According to the results of the confocal laser scanning microscope, the surface roughness of Cu NWs/CF was almost six times that of CF, increasing from 0.28 μm to 1.60 μm (Figure S5). In order to properly understand the formation of the Cu nanowires, we analyzed the XRD patterns of samples at different fabrication stages. As shown in Figure S6a, the typical 020, 021, 040, 130, and 150 crystal planes were detected at 16.5° and 23.4°, 33.7°, 39.6°, and 53.1°, respectively, indicating the formation of Cu(OH)_2_ nanowires after alkaline oxidation [44,45]. After calcination and electro-reduction, the Cu(OH)_2_ crystal planes disappeared, and only copper peaks remained. The XPS method was used to further analyze the composition of the Cu NWs/CF. On the Cu NWs/CF (Figure S6b), the difference between the peaks at 932.3 eV and 952.1 eV was 19.8 eV, which represented the Cu2p_3/2_ and Cu2p_1/2_ peaks of Cu^0^, confirming the existence of elemental copper [46]. There was also a small amount of Cu^2+^ in the sample, which was possibly due to inadequate electroreduction or natural air oxidation. After electroreduction and growth of the Cu nanowires, the purity of the elemental copper was greatly increased (Figure S6c). As can be seen in Figure S7a, the impedance reduced greatly after the growth of Cu nanowires on CF. Moreover, the current of Cu NWs/CF was 1.9 times higher than that of pristine CF at a 1.2 V potential, which indicated that Cu NWs/CF was more conducive to hydrogen generation (Figure S7b).

### 3.2. Hydrogen Generation and Total Nitrogen Removal

**Figure 2:**
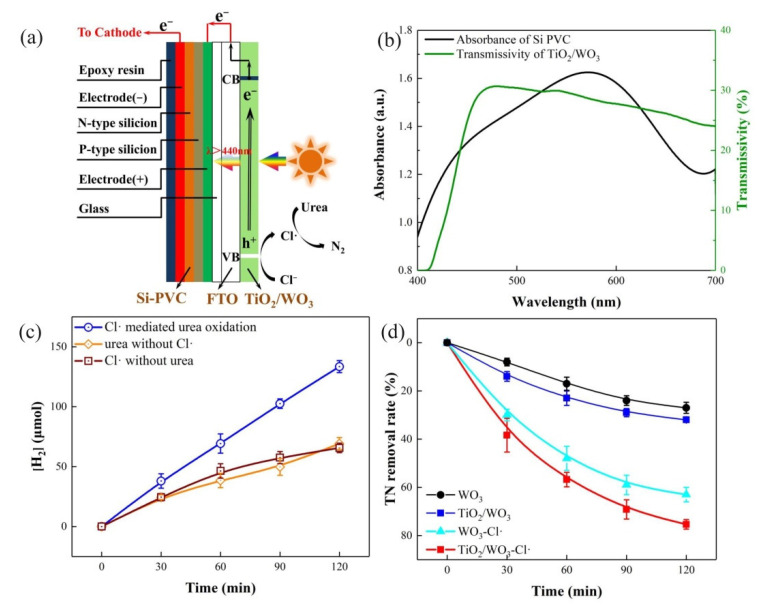
(**a**) Schematic diagram of TiO_2_/WO_3_/Si PVC composite photoanode; (**b**) absorbance of Si PVC and transmissivity of TiO_2_/WO_3_ electrode; (**c**) hydrogen generation under different conditions; (**d**) TN removal rate during a 2 h operation under different conditions. Conditions: 20 mg L^−1^ urea; 50 mmol L^−1^ Na_2_SO_4_; 75 mmol L^−1^ NaCl; solution pH = 7.

Figure 2a shows the schematic diagram of the self-bias composite photoanode and Cu NWs/CF cathode-based PEC system. Cu NWs/CF was employed as the cathode to produce H_2_, and to simultaneously eliminate the over-oxidized nitrate-nitrogen. TiO_2_/WO_3_ was used as the front photoanode material to generate Cl• and HO• for the conversion of urea-nitrogen to N_2_ and the degradation of organic matter, respectively. Si PVC was applied as the rear photoanode to promote the separation of photogenerated charges. As shown in Figure 2b, TiO_2_/WO_3_ utilized sunlight with a wavelength of less than 460 nm, and sunlight with a wavelength of more than 460 nm, which passed through TiO_2_/WO_3_, was utilized by Si PVC. Si PVC was attached to the back of the TiO_2_/WO_3_, and was sealed as a composite photoanode by silicone rubber to fully utilize the sunlight. TiO_2_/WO_3_ produced photogenerated holes and electrons, and the internal electric field provided by Si PVC transferred photo-generated electrons to the Cu NWs/CF cathode, prolonging the life of the photo-generated holes, and promoting the H_2_ production. Synthetic urine, as a test sample that was compounded according to Table S1 (including 0.266 mol L^−1^ urea), was used to investigate the H_2_ generation and TN removal from urine. The contents of the blank group and the treatment group are shown in Table S2. Figure 2c shows the H_2_ generation through using the PEC system with and without Cl•. Hydrogen generation with Cl• is 2.03 times greater than without Cl•, generating hydrogen at 66.71 μmol h^−1^, since the Cl• evolution reaction (ClER) has a kinetic advantage, and Cl• has been verified to promote the oxidation of urea [47]. It should be noted that urea and Cl^−^ are both indispensable for highly efficient H_2_ generation. Without urea, H_2_ generation had a downtrend during the degradation process. The reason for this was that, although Cl• was produced, there was no reactant, such as urea, reacting with Cl•, and the Cl^−^-Cl• cycle could not be realized, which showed the importance of urea. Urea and Cl^−^ cooperate to promote H_2_ production under neutral pH conditions.

Figure 2d shows that the system can also achieve TN removal from urine. The TN removal rate was obviously much slower in the absence of Cl•. The performance of the TiO_2_/WO_3_ photoanode is superior compared to the WO_3_ photoanode, due to better light response and stability (Figure S1). Urea was rapidly and completely degraded with a removal rate of 98.33%, and a reaction rate constant of 0.0359 min^−1^ (Figure S8). The TN removal rate reached 75.3% after a 2 h operation under optimal conditions, with almost only nitrate-nitrogen left, and after a 4 h operation, the TN removal rate reached 90.2% due to a continuous reduction of nitrate-nitrogen on the cathode. The TOC removal rate also can reach 48.4% in 2 h, and 56.0% in 4 h (Figure S9). Figure S10 showed the variation of different nitrogen species during the decomposition of urea. As can be seen, the concentration of nitrite-nitrogen and ammonia-nitrogen was fairly small, whereas the nitrate-nitrogen clearly appeared after a 60 min reaction. As the reaction continued, the concentration of nitrate nitrogen gradually decreased, indicating that most of the nitrogen in the urea had been converted to N_2_. The reason can be attributed to an exhausted conversion of soluble nitrogen species to N_2,_ with the synergistic function of the Cl• radicals and the Cu NWs/CF electrode; this will be further discussed below. Overall, this system achieved excellent TN removal from urine, and simultaneous H_2_ energy recovery under neutral pH conditions.

### 3.3. Influence Factors

#### 3.3.1. Effect of Cathode Type

**Figure 3:**
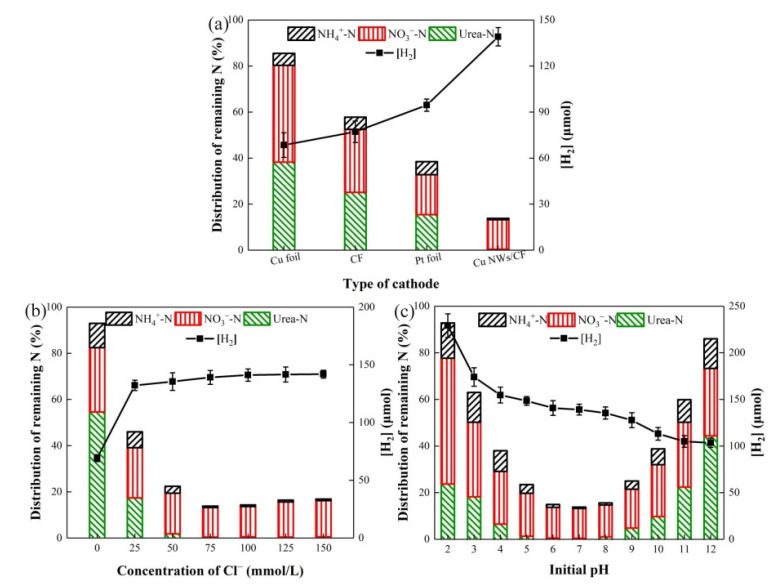
Effect of (**a**) cathode type; (**b**) Cl^−^ concentration; and (**c**) initial pH upon hydrogen generation and TN removal. Conditions: urea 20 mg L^−1^; 50 mmol L^−1^ Na_2_SO_4_.

The function of the cathode in this system is to generate H_2_, and eliminate the trace nitrate produced by the excessive oxidation of urea in the photoanode area. The Cu NWs/CF had an excellent performance, especially concerning H_2_ generation, which was verified by experiments. Figure 3a shows the H_2_ generation and nitrogen removal by using Cu NWs/CF, and three common electrodes (copper foil, CF, and platinum foil electrode), respectively (immersion area of 4 cm^2^). After a 2 h reaction, the H_2_ production rates were 68.47, 77.16, 94.50, and 139.06 μmol, and the TN removal rates were 14.49%, 35.2%, 51.57%, and 75.30% for copper foil, CF, platinum electrodes, and Cu NWs/CF, respectively, suggesting that Cu NWs/CF was significantly better than the other three electrodes. We designed an experiment to compare the reduction rate of nitrate by using four electrodes; Figure S11 shows that the nitrate reduction performance of Cu NWs/CF is obviously better than the other three electrodes. Although the platinum electrode is expensive, it has good nitrogen removal rates and H_2_ generation effects. However, compared with the expensive platinum electrode, the H_2_ generation of Cu NWs/CF increased by 47.15%, and the two-hour TN removal rate also increased from 51.57% to 75.30%. Calculating the cost of platinum electrode versus Cu NWs/CF, the production cost per unit area of Cu NWs/CF is only 0.2% of that of platinum electrode. The reason for the high current density and excellent denitrification effect of the Cu NWs/CF electrodes is the specific surface area and active site of the three-dimensional structure, which greatly accelerates electron transport, and obtains a higher current density. Therefore, under the same conditions, only Cu NWs/CF could achieve rapid TN removal and large amounts of H_2_ generation with low cost.

#### 3.3.2. Effect of Cl^−^ Concentration

The concentration of Cl^−^ directly affects the denitrification of urine and H_2_ generation. Figure 3b shows the proportion of the remaining nitrate-nitrogen, ammonia-nitrogen, and urea-nitrogen, as well as the related hydrogen generation after a 2 h reaction, when the Cl^−^ concentration was 0, 25, 50, 75, 100, 125, and 150 mmol L^−1^, respectively. The result showed that the TN removal rate rapidly increased once Cl^−^ was added, indicating that Cl^−^ played an essential role in the degradation of urea. The most suitable Cl^−^ concentration for TN removal was 75 mmol L^−1^, when urea was completely degraded with the smallest remaining amounts of nitrate-nitrogen and ammonia-nitrogen, and the TN removal rate reached 75.3%. When the Cl^−^ concentration was higher than 75 mmol L^−1^, although urea was completely oxidized, the remaining nitrate-nitrogen increased, which indicated that a denitrification effect would be slightly restrained when Cl^−^ concentration was extremely high. When the Cl^−^ concentration was lower than 75 mmol L^−1^, a part of the urea remained, with large amounts of nitrate and ammonium left. Correspondingly, the H_2_ generation increased by 1.93 times after adding 75 mmol L^−1^ Cl^−^; however, it remains relatively stable with a further increase of Cl^−^ concentration. Considering both the H_2_ generation and TN removal, the optimal Cl^−^ concentration herein is suggested to be 75 mmol L^−1^.

#### 3.3.3. Effect of Initial pH

Solution pH has a significant impact on urine TN removal by affecting the performance of the electrodes and oxidation-reduction reaction in the solution. The H_2_ generation and TN removal effect at pH 2–12 were investigated. The result is shown in Figure 3c. For H_2_ generation, the overall trend was that H_2_ generation increased with the decrease of pH; however, when the solution atmosphere was over-acid, the TN removal effect of urine and the stability of the electrode system was affected. Neutral pH conditions are most conducive to TN removal. When the pH was between 5–8, most of the urea was degraded; in contrast, when the solution pH was over-acid or over-alkaline, part of the urea remained, with large amounts of nitrate-nitrogen and ammonia-nitrogen left behind [48]. under the condition of overacidity, reducibility of the system was motivated by high concentration of H^+^, resulting in an accumulation of ammonium. At the same time, H^+^ competed for electronics with nitrate at the cathode, leading to accumulation of nitrate-nitrogen. Similarly, In the condition of over-alkalinity, the oxidability of the system promoted the formation of nitrate-nitrogen. In addition, the HO• potential was shifted negatively in comparison to Cl•; thus the proportion of HO• increased and the proportion of Cl• decreased, which affected the selective oxidation of ammonium and urea to N_2_, resulting in the accumulation of nitrate-nitrogen. Furthermore, the TiO_2_/WO_3_ and Cu NWs/CF have the best stability at neutral pH. The performance of the TiO_2_/WO_3_ photoanode was affected under the condition of over-alkalinity, which reduced the number of radicals in the system and weakened the degradation of urea. The nanowire fractures of Cu NWs/CF were corroded under the condition of overacidity, with the specific surface area reduced, which affected the reduction of nitrate. Hence, in order to ensure good urine TN removal effect and simultaneous H_2_ generation, a pH of 7 should be the optimal condition, which is consistent with the theme of this work.

### 3.4. Mechanism of Hydrogen Generation and TN Removal

**Figure 4:**
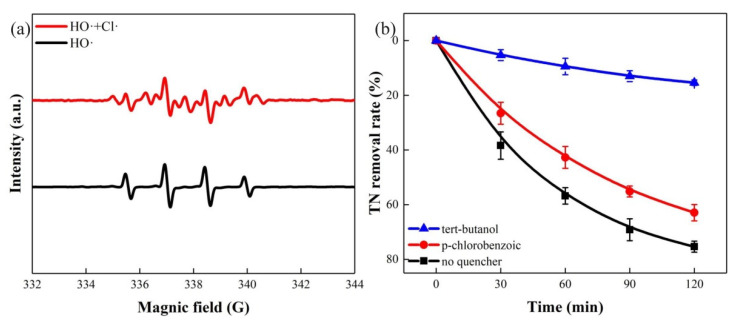
(**a**) EPR spectra of radicals with and without NaCl; (**b**) free radical quenching experiment. Conditions: 20 mg L^−1^ urea; 50 mmol L^−1^ Na_2_SO_4_; 75 mmol L^−1^ NaCl; solution pH = 7.

In order to explore the mechanism of H_2_ generation and urine TN removal in the PEC system, we first examined the free radicals (HO• or Cl•) through an EPR spectra, using 5,5-Dimethyl-1-pyrroline N-oxide (DMPO) as a trapping agent. As can be seen in Figure 4a, four equidistant peaks of DMPO-HO• with an intensity of 1:2:2:1 were observed without Cl^−^, while another seven-line signals corresponding to DMPO-Cl• were detected with Cl^−^ [49]. Afterwards, free radical-quenching experiments were carried out [50]. Two kinds of free radical quencher were used to investigate the function of HO• and Cl• in the reaction process. As is well known, tert-butanol quenches both HO• and Cl•, and p-chlorobenzoic acid only quenches HO•. Therefore, as shown in Figure 4b, after adding 10 mmol L^−1^ tert-butanol into the solution, the removal rate of TN was remarkably reduced to 15.38%. However, the removal rate of TN was still as high as 62.93% when using p-chlorobenzoic acid. The experiment result showed that, when there was only Cl• in the solution, the TN removal effect was still positive, indicating that Cl• can be catalyst of urine denitrification without HO•. On the other hand, when Cl^−^ was not added to the system, a part of urea, nitrate-nitrogen, and ammonia-nitrogen remained in the solution after the reaction (Figure 3b), which further illustrated the strong function of Cl•. Therefore, Cl• plays a pivotal role in the TN removal process.

**Figure 5:**
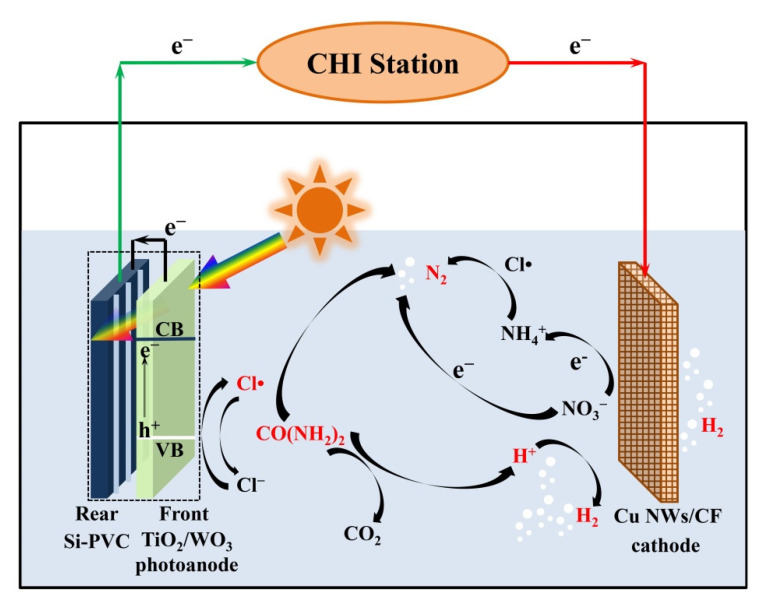
Device schematic diagram and working mechanism of the self-driving PEC system.

According to the research conclusion in this section, the possible TN removal process of urine in this system was inferred (Figure 5). Under the excitation of sunlight, photo-generated hole-electron pairs were generated on the TiO_2_/WO_3_ photoanode (Equation (3)). The photo-generated holes with strong oxidizing properties could promote the generation of free radicals (HO• and Cl•) in the solution (Equations (4)–(6)). Urea molecules were rapidly oxidized and degraded by HO• and Cl•. The difference was that Cl• can selectively oxidize urea/ammonium to N_2_, while HO• oxidized urea/ammonium to NO_3_^−^ (Equations (7)–(10)), which was proven in Figure 3b and Figure 4. The unavoidable NO_3_^−^ produced at the anode was reduced to N_2_ or ammonium in the Cu NWs/CF (Equations (11) and (12)), and ammonium was oxidized to N_2_ in the anode area once again. Repeated cyclic reactions led to the removal of TN.

For the mechanism of H_2_ generation, Cl^−^ and urea collaboratively promoted to produce H_2_. According to the result in Figure 2c, Cl^−^ and urea were both indispensable. In the PEC system, there are oxygen evolution reactions (OERs) and ClERs in the photoanode area. Four electrons are involved in the OER, whereas only one electron is involved in the ClER, which gives the ClER a kinetic advantage. As such, the addition of Cl^−^ could raise the current density and H_2_ generation. However, with a limited amount of Cl^−^, after all of the Cl^−^ was oxidized to Cl•, the current density decreased again. As can be seen in Figure 2c, by simply adding Cl^−^ into the solution, hydrogen production tended to increase in the first half of the reaction, while decreasing in the second half. This problem could be solved by urea, since urea reduced Cl• to Cl^−^, achieving the Cl^−^-Cl• cycle, and promoting H_2_ generation constantly (Equations (13) and (14)). Moreover, the urea oxidation reaction was a process releasing H^+^ (Equation (15) and Figure S12), and the released H^+^ was reduced and utilized at the cathode. At the same time, Cl•, as a nitrogen removal catalyst, degraded urea to purify urine. The cooperation and sufficient presence of Cl^−^ and urea in urine gives PEC urine purification and H_2_ energy recovery a great application potential under neutral pH conditions.

TiO_2_/WO_3_ + hv → h^+^ + e^−^ (3)

h^+^ + OH^−^ → HO• (4)

h^+^ + Cl^−^ → Cl• (5)

HO• + Cl^−^ → Cl• + OH^−^ (6)

16HO• + CO(NH_2_)_2_ → CO_2_ + 2NO_3_^−^ + 9H_2_O + 2H^+^ (7)

8HO• + NH_4_^+^ → NO_3_^−^ + 5H_2_O + 2H^+^ (8)

6Cl• + CO(NH_2_)_2_ + H_2_O → N_2_ + 6Cl^−^ + CO_2_ + 6H^+^ (9)

6Cl• + 2NH_4_^+^ → N_2_ + 6Cl^−^ + 8H^+^ (10)

2NO_3_^−^ + 10e^−^ + 12H^+^ → N_2_ + 6H_2_O (11)

NO_3_^−^ + 8e^−^ + 10H^+^ → NH_4_^+^ +3H_2_O (12)

2H^+^ + 2e^−^ → H_2_ (On Cu NWs/CF) (13)

Cl^−^(+h^+^) → Cl•(+urea) → Cl^−^ (14)

CO(NH_2_)_2_ + H_2_O − 6e^−^ → CO_2_ + N_2_ + 6H^+^ (15)

### 3.5. Application of Actual Urine and Stability of System

**Figure 6:**
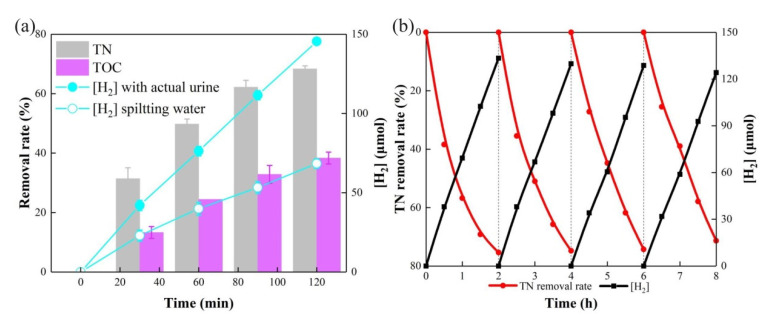
(**a**) TN and TOC removal rate during 2 h operation of actual urine, hydrogen generation of splitting water and actual urine; (**b**) stability test of the PEC system. Conditions: 20 mg L^−1^ urea; 50 mmol L^−1^ Na_2_SO_4_; 75 mmol L^−1^ NaCl; solution pH = 7.

In order to investigate the practical application potential of the PEC system, the application of actual urine and the stability of the system were tested. Actual urine was obtained from 5 healthy volunteers, filtered, and diluted to an appropriate concentration for practical application tests, which contained approximately 0.34 mol L^−1^ urea, and 474.9 mmol L^−1^ Cl^−^. Figure 6a showed that H_2_ generation was 72.73 μmol h^−1^ with actual urine, 2.13 times greater than splitting water, indicating that the presence of actual urine greatly promoted the generation of H_2_, without the addition of extra alkali. The urine TN removal rate reached 68.36% after a 2 h operation under optimal conditions, and after a 4h operation, the TN removal rate reached 84.9% due to a continuous reduction of NO_3_^−^ on the cathode. The TOC removal rate also reached 38.4% in 2 h. It indicated that the system could achieve TN removal from actual urine.

The stability of the system, which is limited by the performance of the photoanode and the physical stability of the cathode, directly determines whether the technology can be applied in practice. As such, the operating stability of the system was tested. The experiment was carried out for 4 consecutive cycles, for a total of 8 h. At the same time, the H_2_ generation and TN removal effect of the anode urea were monitored for 4 cycles. As shown in Figure 6b, in the fourth reaction cycle, the urine TN removal rate still reached 71.34%. Compared with the first cycle, the H_2_ generation in the fourth cycle dropped only by 6.9%. This shows that the stability of the system is excellent, and that this PEC system has great practical application and development potential.

## 4. Conclusions

In this work, we developed a self-driving nano PEC system to rapidly purify urine, and simultaneously recover H_2_ energy from urine in neutral solutions. As the photoanode, TiO_2_/WO_3_ nanosheets generated sufficient Cl• to oxidize the urea. Cu NWs/CF, which had a large specific surface area and great conductivity, was appropriate for the hydrogen evolution reaction. The high selectivity of Cl• sped up the TN removal process. Solar panels allowed for the system to only be driven by solar energy. A total of 75.3% of TN was removed within 2 h, and 90.2% of TN was removed within 4 h. The addition of actual urine doubled the H_2_ generation. This system achieved excellent H_2_ energy recovery and simultaneous TN removal from urine under neutral pH conditions.

## Supplementary Materials

The following are available online at https://www.mdpi.com/article/10.3390/nano11112777/s1, Table S1: The composition of synthetic urine, Table S2: The recipes of sample solution in the blank and treatment group, Figure S1: (a) Chopped LSV curvces of the WO_3_ and TiO_2_/WO_3_ photoanode in 0.1 mol L^−1^ Na_2_SO_4_; (b) photo-response current and stability of WO_3_ and TiO_2_/WO_3_ electrodes, Figure S2: UV–vis absorption spectrum of the TiO_2_/WO_3_ and WO_3_ photoanodes, Figure S3: (a) XRD patterns of FTO, WO_3_, TiO_2_/WO_3_ electrodes; (b) core-level XPS spectra of Ti2p of TiO_2_/WO_3_ electrode, Figure S4: TEM image of Cu nanowire, Figure S5: Surface roughness of CF (a) and Cu NWs/CF (b) using confocal laser scanning microscope, Figure S6: (a) XRD patterns of CF, Cu(OH)_2_ NWs/CF, Cu NWs/CF electrode; (b) core-level XPS spectra of Cu2p of Cu NWs/CF electrode; (c) core-level XPS spectra of Cu2p of CF electrode, Figure S7: (a) Nyquist plots of CF and Cu NWs/CF at 0.6 V potential (vs. Ag/AgCl); (b) LSV curves of CF and Cu NWs/CF, Figure S8: The rate curves and kinetic curves of urea removal in optimal conditions, Figure S9: TN and TOC removal rate during 4h operation, Figure S10: The concentration of ammonia-N, nitrate-N, nitrite-N and urea during the reaction process, Figure S11: Nitrate reduction rate constant of different cathodes, Figure S12: pH value, urea removal rate and H_2_ generation over time during reaction. Refs. [51,52,53] are cited in the Supplementary Materials.
