# Supplementary material for: Efficient Hydrogen Generation and Total Nitrogen Removal for Urine Treatment in a Neutral Solution Based on a Self-Driving Nano Photoelectrocatalytic System
Source: Nanomaterials (Basel). 2021 Oct 20;11(11):2777. doi: 10.3390/nano11112777 (PMC8622695; doi:10.3390/nano11112777)
Supplement: Supplementary file 1 [file nanomaterials-11-02777-s001.zip › nanomaterials-1414639-supplementary.pdf]

nanomaterials

MDPI

Supplementary Materials

Efficient Hydrogen Generation and Total Nitrogen Removal for Urine Treatment in a Neutral Solution Based on a Self-Driving Nano Photoelectrocatalytic System

Pengbo Wang [1], Jinhua Li [1,*], Yang Xu [1], Changhui Zhou [1], Yan Zhang [1], Lina Zha [1], Bo Zhang [1], Jing Bai [1,2] and Baoxue Zhou [1,2,*]

1 Key Laboratory of Thin Film and Microfabrication Technology (Ministry of Education), School of Environmental Science and Engineering, Shanghai Jiao Tong University, Shanghai 200240, China; tianliangjue@sjtu.edu.cn (P.W.); xy0122@sjtu.edu.cn (Y.X.); zhouchanghui-sjtu@sjtu.edu.cn (C.Z.); yan-zhang@sjtu.edu.cn (Y.Z.); zhalina_V5@sjtu.edu.cn (L.Z.); zhangb229@sjtu.edu.cn (B.Z.); bai_jing@sjtu.edu.cn (J.B.)

2 Shanghai Institute of Pollution Control and Ecological Security, Shanghai 200092, China

* Correspondence: lijinhua@sjtu.edu.cn (J.L.); zhoubaoxue@sjtu.edu.cn (B.Z.)

Preparation of WO_3 Photoanode

WO_3 was prepared by hydrothermal method. The FTO was ultrasonically cleaned in deionized water, ethanol, and acetone for 1 h respectively for later use. The preparation process of the tungsten precursor solution was as follows: Firstly, 1 g of ammonium metatungstate was added to 93 mL of water and stirred for 1h; Then, 2 mL of concentrated hydrochloric acid was added and stirred for 0.5 h; Finally, 4ml of hydrogen peroxide was added and stirred for 0.5 h. Placed the cleaned FTO face-down in a 75 mL reactor and immersed it in the tungsten precursor solution. Placed the reactor in an oven at 160 °C for 240 min. Then the electrode was annealed at 500 °C for 2 h at a heating rate of 1 °C min^{-1} in a muffle furnace air atmosphere.

Table S1. The composition of synthetic urine.

	**D. McCurdy [1]**	**Daniella Saetta [2]**	**Hannah Ray [3]**	**This paper**
Species	C (mol L^{-1})	C (mol L^{-1})	C (mol L^{-1})	C (mol L^{-1})
Urea ($CO(NH_2)_2$)	0.266	0.25	0.25	0.266
NaCl	0.040	0.044	0.044	-
Na_2SO_4	0.030	0.015	0.015	0.050
KCl	0.046	0.040	0.040	-
KH_2PO_4	-	-	-	0.025
$MgCl_2·6H_2O$	0.003	0.004	0.004	-
$MgSO_4$	-	-	-	0.004
NH_4Cl	0.020	-	-	-
NH_4HCO_3	-	-	-	0.020
NaH_2PO_4	0.020	0.020	0.020	0.020
$CaCl_2·2H_2O$	-	0.004	0.005	-

Table S2. The recipes of sample solution in the blank and treatment group.

	Urea without Cl•	Cl• without urea	Cl• mediated urea oxidation
Urea (mg L^{-1})	20	-	20
NaCl (mmol L^{-1})	-	75	75
Na_2SO_4 (mmol L^{-1})	50	50	50
KH_2PO_4 (mmol L^{-1})	0.5	0.5	0.5
$MgSO_4$ (mmol L^{-1})	0.08	0.08	0.08
NH_4HCO_3 (mmol L^{-1})	0.4	0.4	0.4
NaH_2PO_4 (mmol L^{-1})	0.4	0.4	0.4

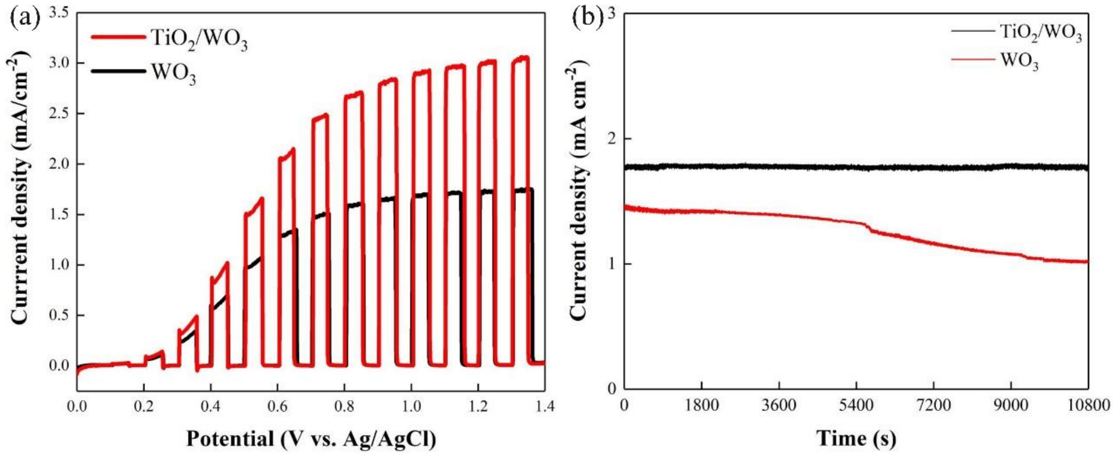

Figure S1. (**a**) Chopped LSV curvces of the WO_3 and TiO_2/WO_3 photoanode in 0.1 mol L^{-1} Na_2SO_4; (**b**) photo-response current and stability of WO_3 and TiO_2/WO_3 electrodes with conditions: at 0.6 V vs. Ag/AgCl) in 0.1 mol L^{-1} Na_2SO_4, pH 7, under simulated solar light (100 mW cm^{-2}).

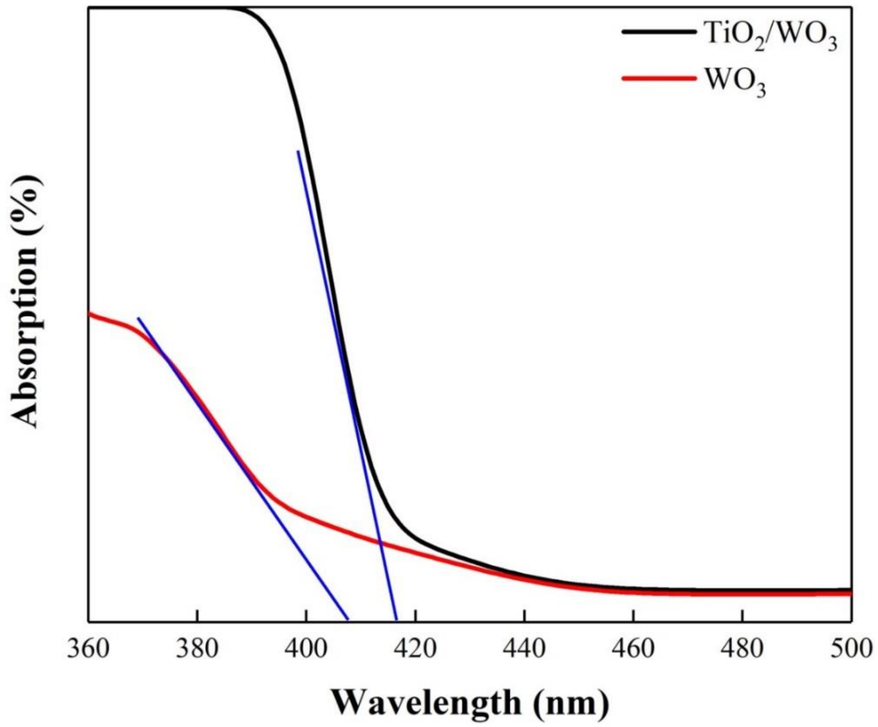

Figure S2. UV–vis absorption spectrum of the TiO_2/WO_3 and WO_3 photoanodes.

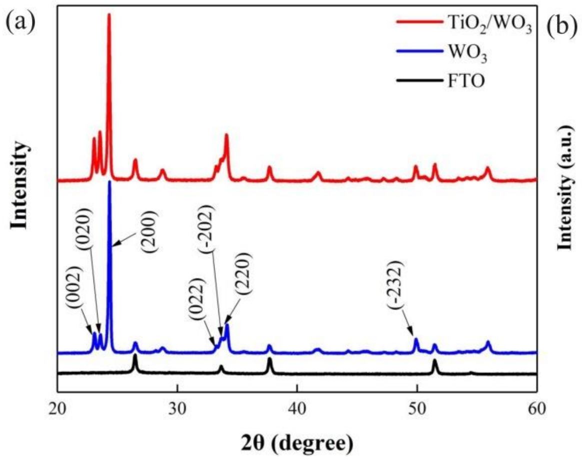

Figure S3. (**a**) XRD patterns of FTO, WO_3, TiO_2/WO_3 electrodes; (**b**) core-level XPS spectra of Ti2p of TiO_2/WO_3 electrode.

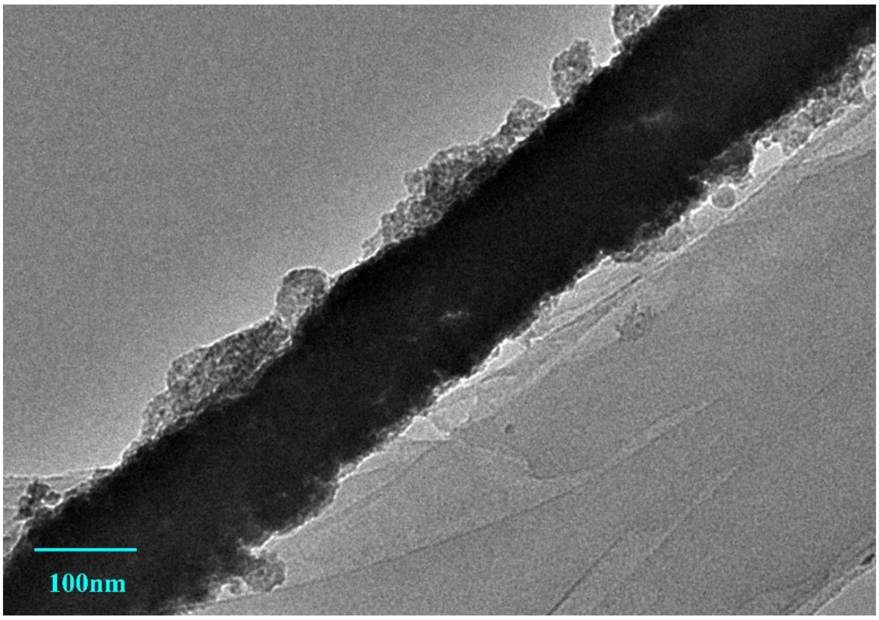

Figure S4. TEM image of Cu nanowire.

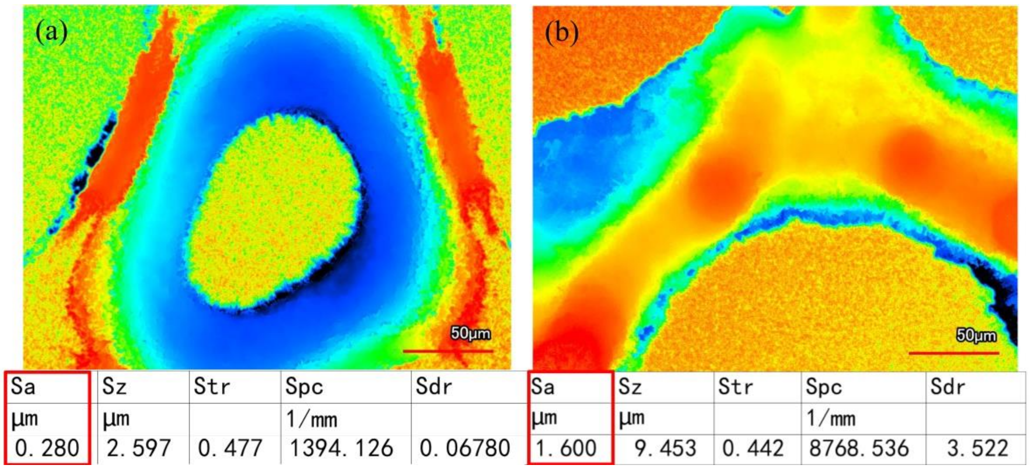

Sa	Sz	Str	Spc	Sdr
μm	μm		1/mm	
0. 280	2. 597	0. 477	1394. 126	0. 06780

Sa	Sz	Str	Spc	Sdr
μm	μm		1/mm	
1. 600	9. 453	0. 442	8768. 536	3. 522

Figure S5. Surface roughness of CF (**a**) and Cu NWs/CF (**b**) using confocal laser scanning microscope.

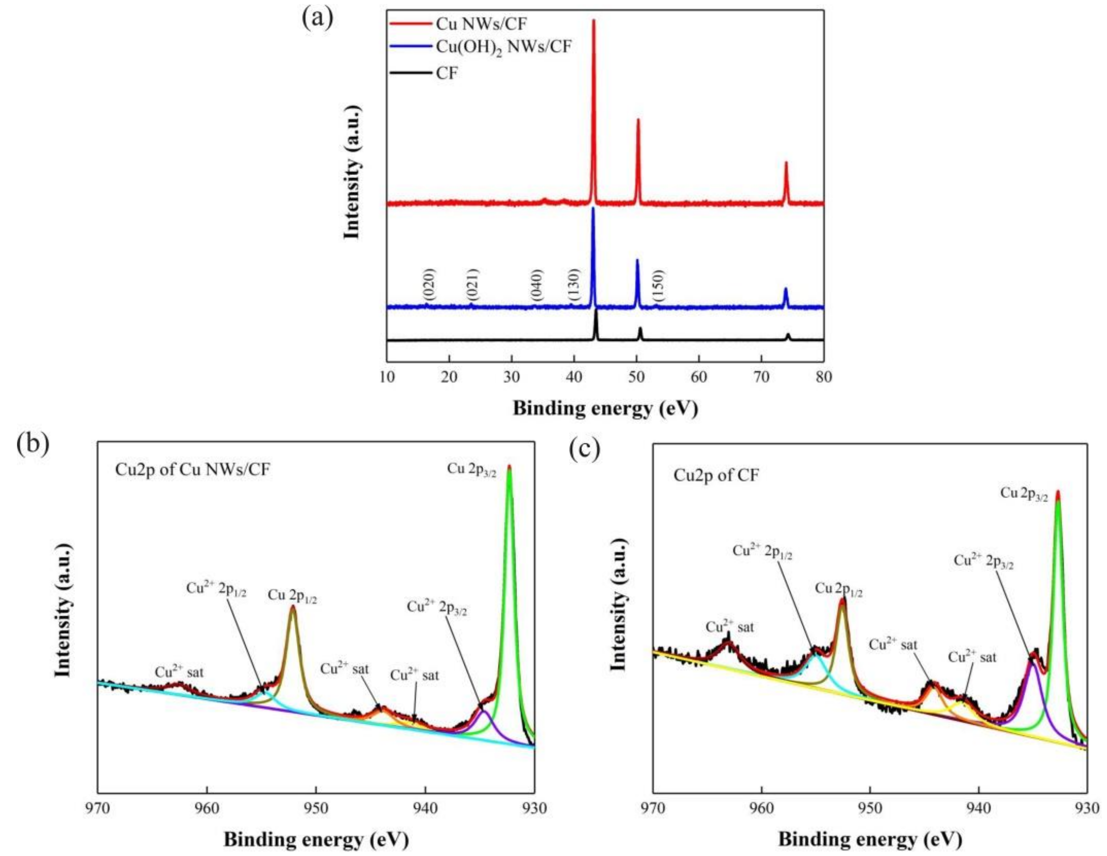

Figure S6. (**a**) XRD patterns of CF, $Cu(OH)_2$ NWs/CF, Cu NWs/CF electrode; (**b**) core-level XPS spectra of Cu2p of Cu NWs/CF electrode; (**c**) core-level XPS spectra of Cu2p of CF electrode.

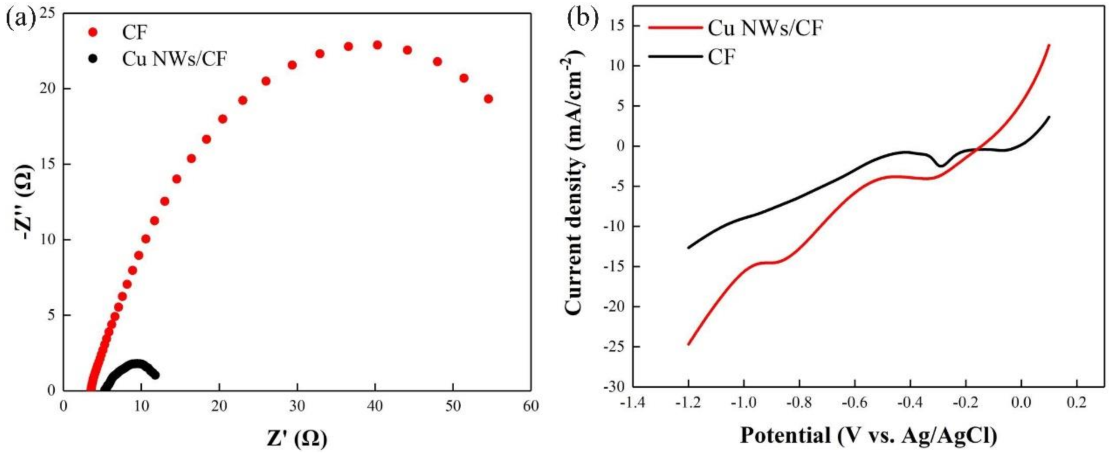

Figure S7. (**a**) Nyquist plots of CF and Cu NWs/CF at 0.6 V potential (vs. Ag/AgCl); (**b**) LSV curves of CF and Cu NWs/CF in 0.1 mol L^{-1} Na_2SO_4.

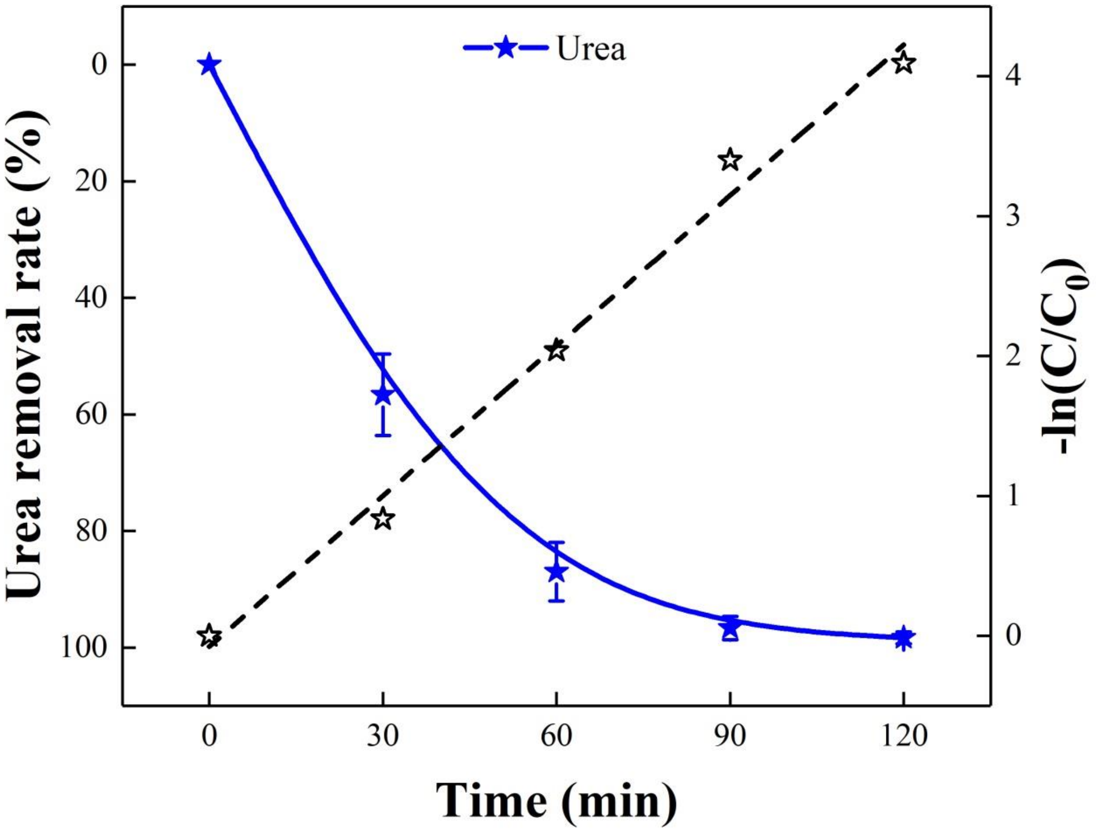

Figure S8. The rate curves and kinetic curves of urea removal in optimal conditions. Conditions: urea 20 mg L^{-1}, solution pH=7, 75 mmol L^{-1} NaCl , 50 mmol L^{-1} Na_2SO_4.

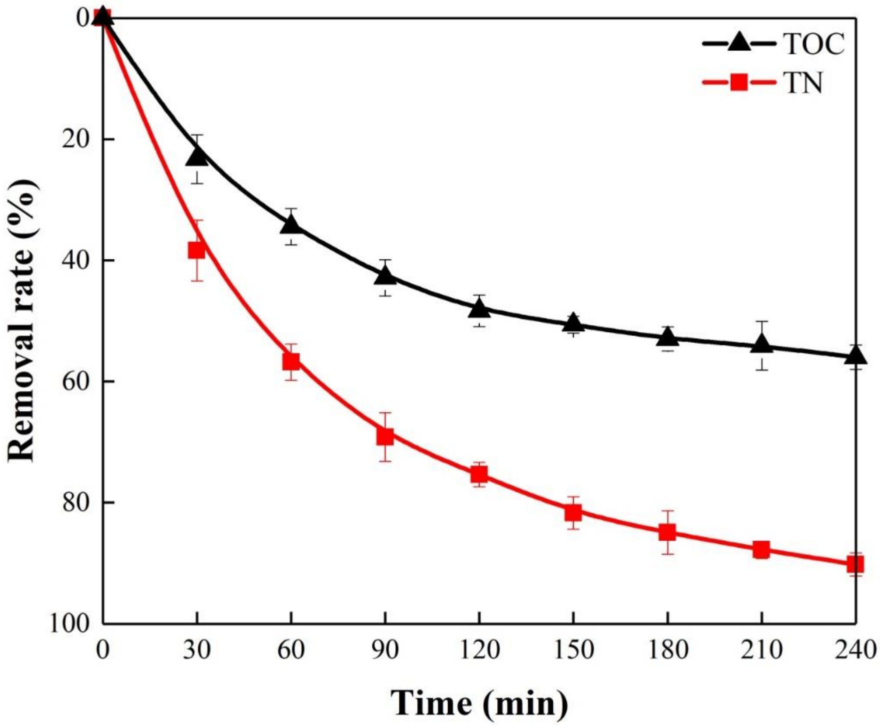

Figure S9. TN and TOC removal rate during 4h operation. Conditions: 20 mg L^{-1} urea, 50 mmol L^{-1} Na_2SO_4, 75 mmol L^{-1} NaCl, solution pH=7.

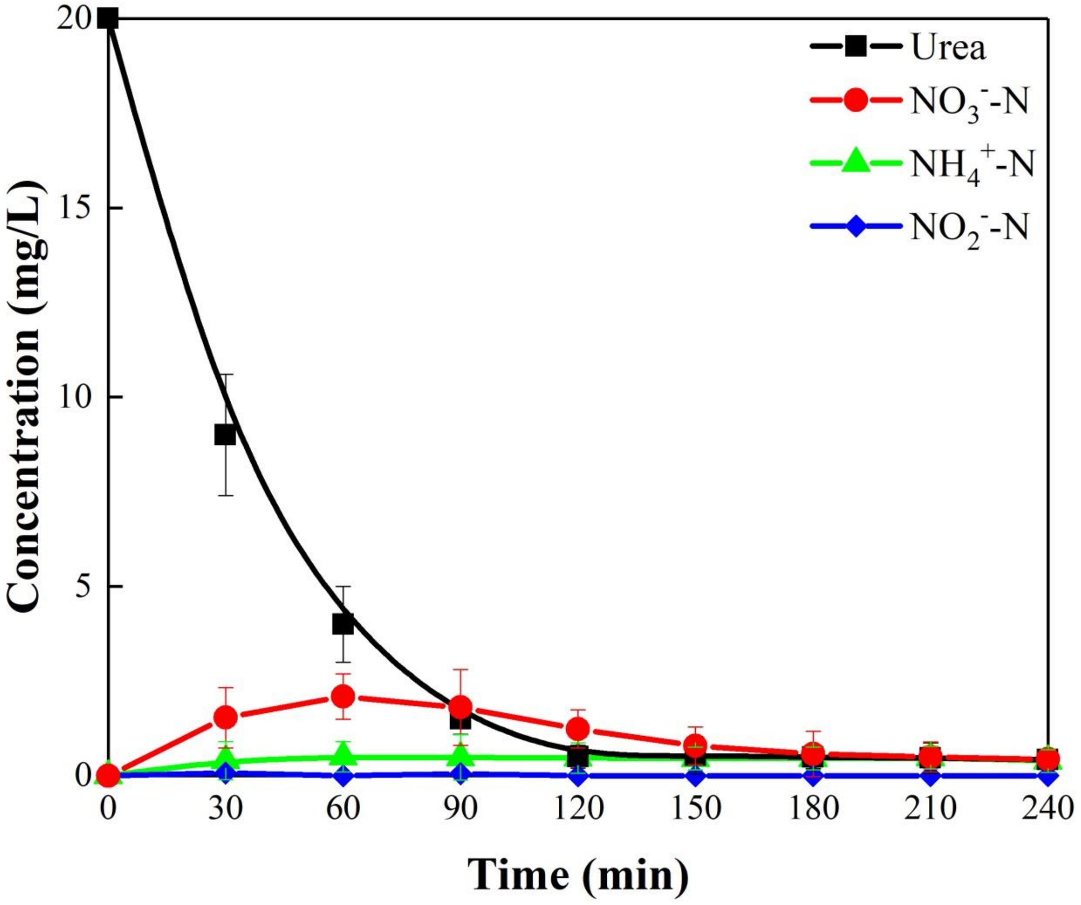

Figure S10. The concentration of ammonia-N, nitrate-N, nitrite-N and urea during the reaction process. Conditions: urea 20 mg L^{-1} , solution pH=7, 75 mmol L^{-1} NaCl , 50 mmol L^{-1} Na_2SO_4.

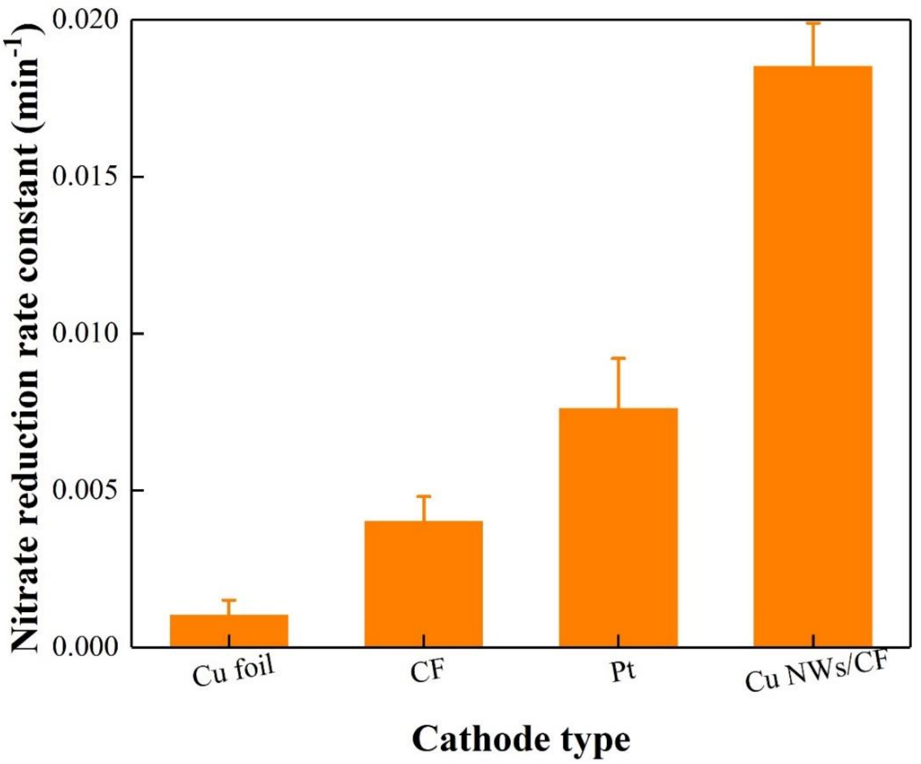

Figure S11. Nitrate reduction rate constant of different cathodes. Conditions: 30mg L^{-1} NO_3^--N, 50mmol L^{-1} Na_2SO_4, 75mmol L^{-1} NaCl, initial pH 7.

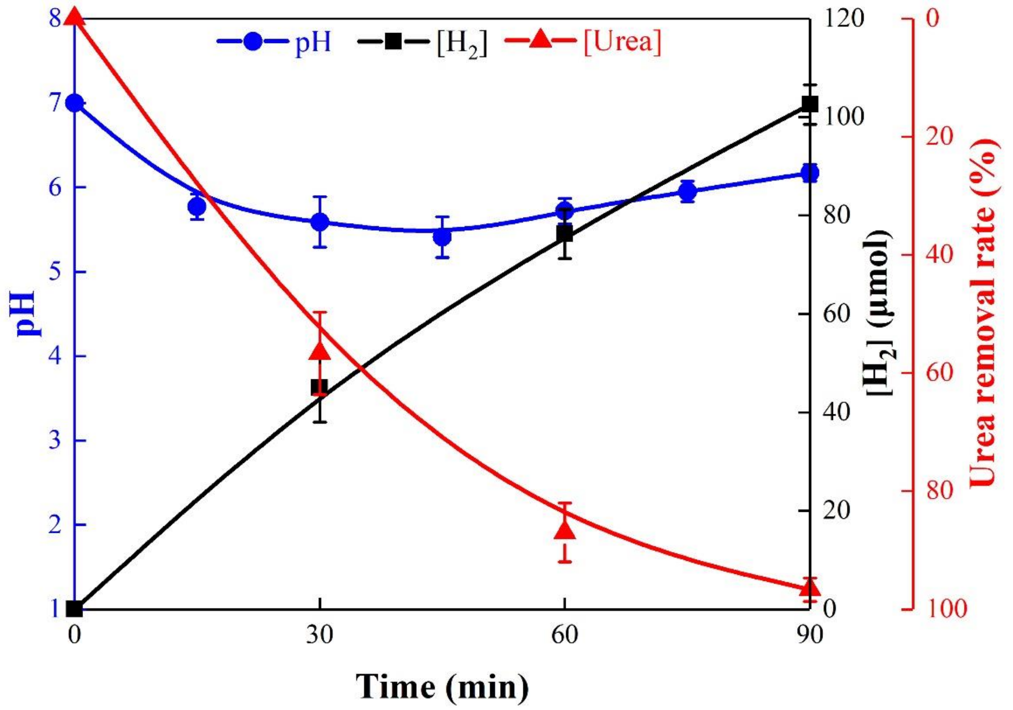

Figure S12. pH value, urea removal rate and H_2 generation over time during reaction. Conditions: 20 mg L^{-1} urea, 50 mmol L^{-1} Na_2SO_4, 75 mmol L^{-1} NaCl, initial solution pH=7.

References

1. McCurdy, D.; Lin, Z.; Inn, K.; Bell III, R.; Wagner, S.; Efurd, D.; Steiner, R.; Duffy, C.; Hamilton, T.; Brown, T. Second interlaboratory comparison study for the analysis of 239Pu in synthetic urine at the microBq (-100 aCi) level by mass spectrometry. *J. Radioanal. Nucl. Chem*. **2005**, *263*, 447–455.
2. Saetta, D.; Boyer, T.H. Mimicking and inhibiting urea hydrolysis in nonwater urinals. *Environ. Sci. Technol*. **2017**, *51*, 13850–13858.
3. Ray, H.; Perreault, F.; Boyer, T.H. Urea recovery from fresh human urine by forward osmosis and membrane distillation (FO–MD). *Environ. Sci. Water Res. Technol.*, **2019**, *5*, 1993–2003.
